# Endocrine Sequelae of Mild Traumatic Brain Injury in Patients Admitted to the Emergency Department: A 12-Month Study

**DOI:** 10.3390/diagnostics16060955

**Published:** 2026-03-23

**Authors:** Maria Kałas, Mariusz Siemiński, Ewelina Stępniewska

**Affiliations:** Department of Emergency Medicine, Medical University of Gdansk, 80-210 Gdansk, Poland; mariusz.sieminski@gumed.edu.pl (M.S.); e.stepniewska@gumed.edu.pl (E.S.)

**Keywords:** euthyroid sick syndrome, low triiodothyronine, mild Traumatic Brain Injury, post-traumatic hypopituitarism, prolactin

## Abstract

**Background/Objectives:** Over the last two decades, there has been a substantial change in the understanding of post-traumatic hypopituitarism (PTHP), which is no longer regarded as a marginal phenomenon. Clinical manifestations of pituitary hormone deficiency are frequently nonspecific, with fatigue and cognitive dysfunction predominating. Given that head injuries currently constitute a global burden for healthcare systems, the aim of the present study was to determine whether self-reported post-mild traumatic brain injury (mTBI) symptoms that may indicate hypopituitarism reflect true pituitary insufficiency or are attributable to other hormonal aberrations. The study aimed to assess the relationship between self-reported symptoms of PTHP and hormonal test results following mTBI. Setting: Patients were recruited from a tertiary trauma center Emergency Department (ED) in northern Poland from January 2023 to October 2025. Participants: The participants were adult (18 > y.o.) individuals with mTBI who met the inclusion criteria. Design: This was a prospective cohort study. During their post-head injury admission to the ED, patients had a blood sample taken. The procedure was repeated consecutively after 3, 6 and 12 months. After 6 and 12 months, patients were asked to complete a questionnaire. **Methods**: Pituitary and thyroid hormones were measured using the chemiluminescence immunoassay method and the heterogenous immunochemiluminescence method. The questionnaire used, *Questionnaire for the Assessment of Symptoms of Anterior Pituitary Insufficiency in Patients After Mild Traumatic Brain Injury (mTBI) Hospitalized in the Emergency Department*, was designed for the purposes of this study. **Results:** Self-reported symptoms suggestive of anterior pituitary dysfunction following mTBI were not confirmed by laboratory assessment of pituitary hormones. However, after 6 months, a statistically significant correlation was found between the number of reported symptoms and prolactin levels (ρ = 0.730; *p* = 0.0013), whereas after 12 months a downward trend in free triiodothyronine (fT3) levels was observed compared with the baseline. **Conclusions:** Persistent symptoms reported by patients following mTBI at 6 and 12 months, particularly fatigue and impaired concentration, showed statistical associations with prolactin levels at 6 months and lower fT3 levels at 12 months. These findings reflect correlations identified in the statistical analysis and do not support inferences regarding causality or the presence of true PTHP.

## 1. Introduction

Traumatic brain injury (TBI) is defined as non-congenital and non-degenerative brain injury sustained as a result of an external force, which may lead to transient or permanent neurological impairment [1,2]. Traditionally, brain injuries are classified according to the score obtained on the Glasgow Coma Scale (GCS) during post-traumatic assessment as mild (13–15 points), moderate (9–12 points), or severe (8 points or less) [3,4]. The most common type is mTBI, constituting 80% to 90% of all cases [1,5]. Consistent with its designation, mTBI has traditionally been viewed as a benign injury with no expected long-term consequences. However, advances in neuroimaging, as well as in the understanding of molecular and biochemical processes occurring in the injured brain, undoubtedly necessitate a re-evaluation of this assumption, since current evidence demonstrates that TBIs, even mild ones, may well be associated with numerous long-term, persistent sequelae [6,7], and the concept of TBI as a single injury event with time-limited recovery is no longer viable [8]. Moreover, current data suggest that up to 50% of patients after mTBI do not return to their pre-illness condition [5].

One of the consequences of TBI that has gained increasing research attention over the past 25 years is PTHP. Although the first case was described over 100 years ago, in 1918, it was considered a marginal phenomenon until the early 2000s [9]. Studies conducted since then, however, indicate that its prevalence is substantially higher than previously assumed [9]. The estimated occurrence rate of PTHP varies across studies, ranging from 15% to 60% in adults and up to 42% in children and adolescents [10]. Thus, assuming an estimated global incidence of approximately 50–60 million traumatic brain injuries per year [5], even the most conservative prevalence estimates imply that millions of individuals may be potentially affected by this long-term sequela of injury. Undiagnosed and untreated PTHP may lead to impaired quality of life and place a significant financial burden on healthcare systems [11].

The postulated etiology of PTHP involves an overlap between primary brain injury (direct mechanical impact) and secondary brain injury mechanisms [12,13]. Primary, mechanical injury may lead to damage of the pituitary stalk, the gland itself or the supplying hypophyseal portal veins [12,14]. Secondary insult results from pathophysiological processes following TBI (e.g., hypoxia, ischemia, oedema, hemorrhage, and alterations in cerebral metabolism) [12,15,16]. Finally, the concept of post-traumatic neuroinflammation involving, among other things, tanacytes [17], has gained much attention in recent years. As a consequence of the inflammatory cascade, an autoimmune response is triggered, resulting in the appearance of auto-antibodies: anti-pituitary antibodies (APAs) and anti-hypothalamic antibodies (AHAs) [18].

As indicated above, reported rates of PTHP differ between studies, which may be explained by, among other factors, the use of dynamic testing and the inclusion of patients representing all three brain injury severity categories [19]. On the whole, most studies consistently indicate that the somatotropic axis is the most commonly impaired system in the long-term course following TBI [13]. Accurate diagnosis of PTHP remains difficult because the manifestations of pituitary insufficiency are nonspecific and may overlap with symptoms typical of post-concussion syndrome, which further hinders diagnostic evaluation [20]. The exact clinical phenotype depends on the extent and number of disrupted hormonal pathways [21]. Nevertheless, the most commonly reported symptoms associated with PTHP are fatigue, generalized weakness, and cognitive impairment. Although these symptoms are part of the clinical spectrum of growth hormone deficiency (GHD), they lack specificity and, in patients with traumatic brain injury, may result from multiple other factors and mimic and/or overlap with post-concussion syndrome.

The accurate diagnosis of PTHP, and GHD in particular, is methodologically challenging and depends on dynamic stimulation tests, which are neither economically viable nor feasible for universal application in all patients reporting chronic fatigue following traumatic brain injury. This limitation is reflected in the available literature, as studies utilizing stimulation testing report a markedly lower prevalence of GHD compared with studies in which such tests were not performed [13]. This leads to certain discrepancies, as on the one hand the current literature reports a relatively frequent occurrence of PTHP, while on the other hand clinicians who routinely care for patients after head injury or with endocrine disorders do not observe such a substantial number of cases [22]. There is also no clear consensus in the existing literature regarding the prevalence of PTHP across different levels of traumatic brain injury severity. Although PTHP is generally reported more frequently in patients with severe TBI, Schneider et al. suggested that this association may not apply to mild or moderate injuries, as a higher prevalence of PTHP was observed in patients with mTBI [23].

Therefore, the authors of the present study aimed to investigate whether, in patients with mTBI—the most common form of brain injury from an epidemiological perspective—reported symptoms such as increased fatigue, reduced concentration, and cognitive impairment, which also fall within the post-concussion symptom spectrum, are associated with post-traumatic pituitary dysfunction or may instead be related to other hormonal disturbances.

## 2. Methods

Study setting: This was a prospective single-center study performed in a Polish ED that is a part of a University Hospital and operates as a tertiary referral center. The annual admission rate to the ED is around 38,000 patients.

Patients: Inclusion criteria for the study were as follows: age > 18 years old, meeting the American Congress of Rehabilitation Medicine’s (ACRM) definition of mTBI [24] and having no acute intracranial findings on initial computed tomography (CT). Exclusion criteria were as follows: pregnancy, abnormal post-injury neuroimaging findings, hospitalization > 48 h due to TBI, age < 18 y.o., and lack of informed consent. Patients were recruited from January 2023 until October 2025.

Clinical data: The following demographic and clinical data were collected for each patient: age, sex, and past medical history concerning endocrinological diseases.

Clinical scales: Assessment of patient-reported symptoms of hypopituitarism was performed using a questionnaire developed specifically for the purposes of the present study: *Questionnaire for the Assessment of Symptoms of Anterior Pituitary Insufficiency in Patients After Mild Traumatic Brain Injury (mTBI) Hospitalized in the Emergency Department.* This questionnaire was based on an article by Mele et al. [17] and consists of 18 items addressing symptoms related to dysfunction of the different pituitary axes. The full version of this questionnaire is available in File S1.

Laboratory assessment: During the examination, the following laboratory assessments were carried out in the patients:

- Hormones: free Triiodothyronine (fT3), free Thyroxine (fT4), Thyroid-Stimulating Hormone (TSH), Growth Hormone (GH), Insuline-like Growth Factor 1 (IGF-1), Prolactine (PRL), and Adrenocorticotropic Hormone (ACTH). Reference ranges: TSH: 0.35–4.94 uU/mL; fT3: 1.78–7.07 pmol/L; fT4: 9.01–19.05pmol/L; GH: <8 ng/mL; IGF-1: 89–290 ng/mL; PRL: 108.8–557.1 mU/L; ACTH: <46pg/mL
- Gonadotropic hormones were initially also assessed. However, due to the small size of the study cohort and the relatively high prevalence of confounding factors that could have compromised the scientific validity of the collected data—including uncertain menopausal status, use of hormonal contraception, testosterone replacement therapy, and preparation for gender-affirming treatment—these data were excluded from the final analysis. Consequently, the scope of the study was restricted to hormones directly associated with symptoms that may mimic post-concussion syndrome, such as cognitive impairment, fatigue, and related mood fluctuations.

Study protocol: The following visits were pre-scheduled for each patient, along with the procedures performed at each visit (they are presented in Table 1).

### Statistical Analysis

Data quality was verified by removing duplicate patient records and standardizing missing questionnaire entries. Values reported as below the detection limit (e.g., <x) were conservatively converted to x. Normality was assessed using the Shapiro–Wilk test (*n* ≥ 3) for baseline, follow-up, and paired differences. Changes over time were analyzed using paired *t*-tests or Wilcoxon signed-rank tests as appropriate; variables with fewer than three paired observations were not interpreted. Associations between symptoms and laboratory parameters were assessed using Spearman’s rank correlation, while group comparisons were performed using Welch’s *t*-test or the Mann–Whitney U test. All tests were two-sided with a significance level of α = 0.05; raw *p*-values were reported, with false discovery rate correction (Benjamini–Hochberg) applied where appropriate.

The analyses were performed in the Python environment, version 3 (Python Software Foundation, Beaverton, OR, USA), using the following libraries: pandas (pandas-dev, open-source, available at https://pandas.pydata.org); SciPy—the scipy.stats module (The SciPy Community, open-source, https://scipy.org); statsmodels (the statsmodels development team, open-source, https://www.statsmodels.org); and openpyxl (open-source, https://openpyxl.readthedocs.io)—used for saving and organizing results in Excel spreadsheets. Additional calculations and table preparation were carried out in Microsoft Excel (Microsoft Corporation, Redmond, WA, USA).

## 3. Results

**Figure 1:**
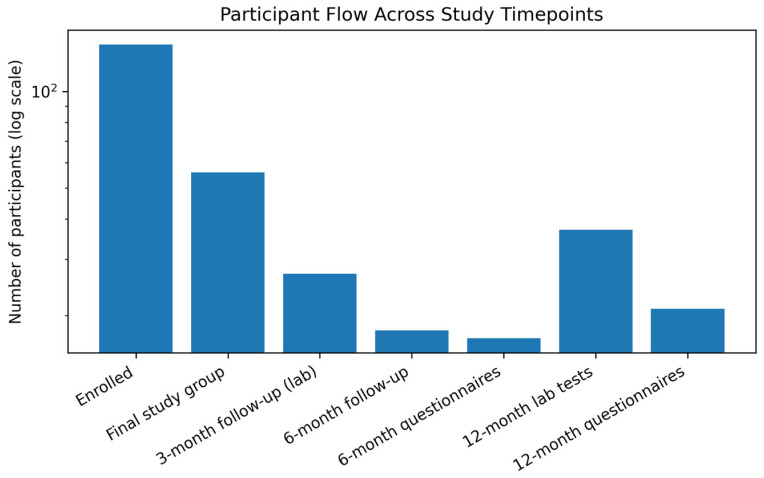
Distribution of patients across different stages of the study.

Patients were recruited from January 2023 until October 2025. A total of *140 individuals were enrolled in the study. However, in the course of the study, eight participants were excluded (five due to traumatic lesions identified on CT imaging, one due to pregnancy, one due to death, and one due to a subsequent severe head injury). Furthermore, 20 participants were excluded because of incomplete baseline data.* Ultimately, the study group consisted of 56 participants. A total of 27 participants attended the laboratory follow-up at 3 months. At 6 months, 18 participants underwent follow-up, of whom 17 completed the questionnaire. After 12 months, 37 follow-up laboratory tests were performed, and 21 questionnaires were completed. The flow and characteristics of the patients are presented in Figure 1 and Table 2.

**Figure 2:**
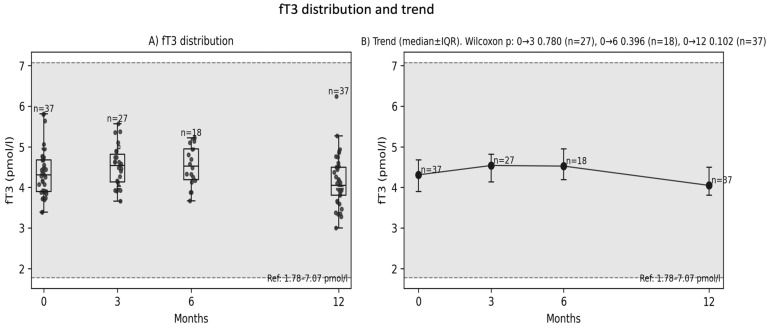
Distribution of fT3 levels post-injury (**A**) and changes in the median fT3 at 3, 6 and 12 months (**B**).

**Figure 3:**
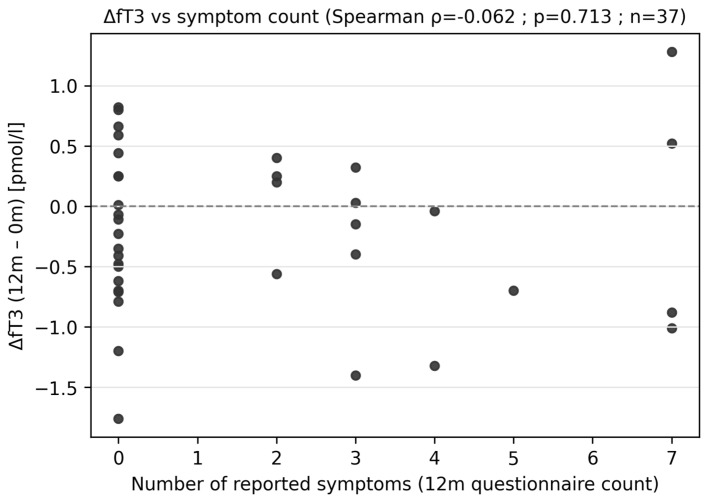
Changes in fT3 levels vs. number of reported symptoms at 12 months.

The 12-month analysis revealed a trend toward decreased fT3 levels (Figure 2A,B); however, the result did not reach statistical significance (paired *t*-test; *p* ≈ 0.0785; *n* = 37; median Δ = −0.150; alternatively, Wilcoxon test *p* ≈ 0.104). No association was found between the change in fT3 after 12 months and the severity of complaints reported in the questionnaire (Figure 3) (ΔfT3 vs. number of complaints Q_count: rho ≈ −0.064; *p* ≈ 0.705), nor were there differences in ΔfT3 between participants with and without complaints (*p* ≈ 0.931).

After 6 months, difficulty concentrating was the most frequently reported symptom, whereas after 12 months, increased fatigue was reported most often (Table 3). Assessment of symptoms and their frequency was performed using a questionnaire designed by the authors for the purpose of this study (the questionnaire is available in File S1).

**Figure 4:**
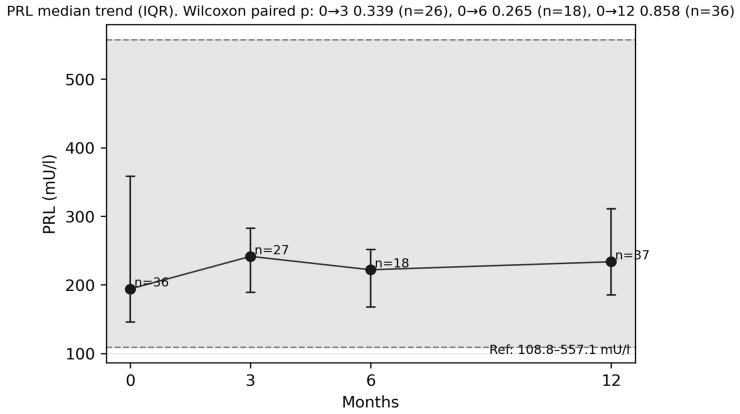
Prolactin median trends at 3, 6 and 12 months.

**Figure 5:**
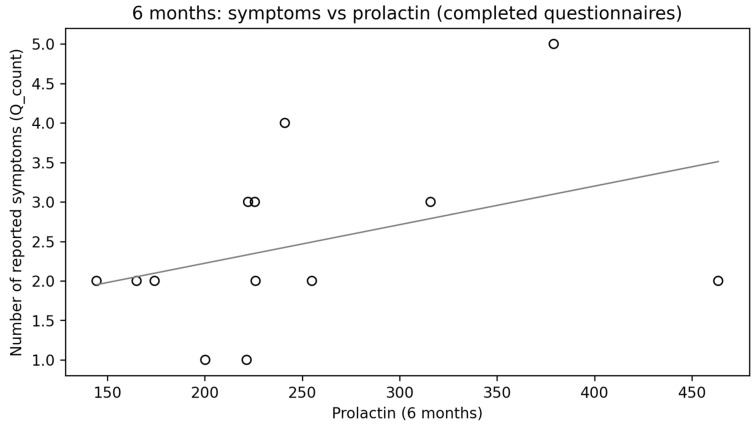
Symptoms vs. prolactin at 6 months.

Although no statistically significant change in prolactin levels was observed after 6 months (Figure 4) compared with baseline values, a strong, positive, and statistically significant correlation was found between the number of reported symptoms and prolactin levels (ρ = 0.730; *p* = 0.0013) (Figure 5). After applying FDR correction, the result remained statistically significant (p_FDR = 0.0173).

**Figure 6:**
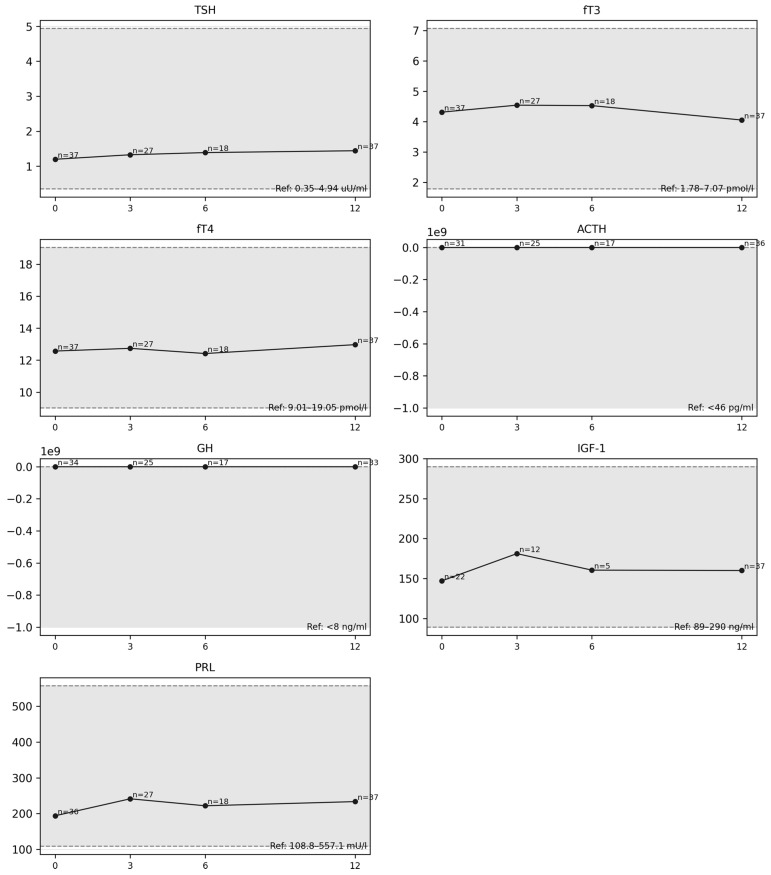
Hormonal changes median values at 3, 6 and 12 months.

No statistically significant changes were found in the analysis of the remaining hormones at 3, 6 and 12 months (Figure 6). However, it should be emphasized that only single hormone measurements were performed in this study, rather than dynamic testing. This may have limited the detection of subtle abnormalities, particularly within the corticotropic and somatotropic axes. Furthermore, the relatively small sample sizes at latter follow-up stages and the use of non-validated questionnaires may have influenced the observed hormone–symptom associations. Moreover, the authors would like to emphasize that the findings of the present study reflect correlations identified in the statistical analysis and do not support inferences regarding causality or the presence of true PTHP.

## 4. Discussion

The occurrence of neuroendocrine disturbances as a response to injury or severe illness is well documented [25,26]. Some endocrine abnormalities, such as glucocorticoid deficiency, if unrecognized and untreated, can have life-threatening consequences in the acute phase [2].

Insufficiency of other hormones may be of prognostic relevance, influencing recovery and subsequent quality of life [11,27].

Thyroid hormones play a crucial role in brain development and are essential for its proper functioning, including the regulation of neuronal plasticity processes, stimulation of angiogenesis and neurogenesis, as well as modulation of cytoskeletal dynamics and intracellular transport processes [28,29,30,31]. Even though the thyrotropic axis, due to the central location of thyrotrope cells within the anterior pituitary, is less frequently damaged after head trauma than the somatotropic or gonadotropic axes [32], numerous studies involving patients after traumatic brain injury have demonstrated abnormalities in thyroid hormone levels [33,34,35]. Consequently, the impact of thyroid hormones on post-TBI recovery and neurological outcome has already been the subject of various investigations [36,37,38,39,40], in which their beneficial effect on functional restoration has been proved. Aberrations in thyroid hormone levels were first described in the 1970s, when critically ill patients—regardless of the underlying etiology—were observed to exhibit low serum concentrations of fT3, or both fT3 and free fT4, in the presence of normal TSH levels. This phenomenon is now referred to as euthyroid sick syndrome (ESS) or non-thyroidal illness syndrome (NTIS) [41]. Since that time, transient disturbances in thyroid hormone parameters have been considered a part of the physiological stress response [9,42] and a characteristic element of the laboratory manifestations of acute illness. However, the present study appears to suggest that fluctuations in fT3 levels may also occur long after head injury, thereby potentially affecting the recovery process.

As was mentioned above, in the majority of studies conducted to date with long-term follow-up, chronic impairment of the pituitary–thyroid axis has been observed less frequently than dysfunction of the somatotropic or gonadotropic axes [2]. In this context, the findings of a recent study conducted exclusively in female athletes who sustained mild TBI are particularly noteworthy. In that cohort, central hypothyroidism was the most commonly diagnosed endocrine abnormality [43]. Notably, most previous investigations of PTHP predominantly included male populations [44], whereas in our study women constituted the majority of participants at all follow-up timepoints. It is therefore conceivable that in women the pituitary–thyroid axis may be more susceptible to post-traumatic injury, suggesting that strategies for post-traumatic monitoring of pituitary dysfunction should also take biological sex into account. The hypothesis of increased susceptibility of the female organism to deviations from physiological homeostasis is supported by a study conducted in a large cohort of women which examined the relationship between trauma exposure, post-traumatic stress disorder (PTSD), and thyroid dysfunction. The authors demonstrated that PTSD was associated with an increased risk of hypothyroidism in a dose-dependent manner. Heightened clinical awareness of thyroid dysfunction may therefore be particularly important in women with PTSD [45]. Therefore, future research in female patients following mTBI appears warranted, with a special focus on fT3 level fluctuations.

No post-traumatic growth hormone deficiency was identified in any patient in our study. Importantly, these findings should be interpreted with caution given that only single hormone measurements were performed instead of dynamic testing, and the groups assessed at the 6- and 12-month follow-ups were relatively small. In the 12-month follow-up cohort, two participants had baseline GH levels below the reference range. However, at the 12-month assessment, GH concentrations below the reference range were identified in three different participants. In all of these individuals, IGF-1 levels remained within the normal range. These findings underscore the importance of appropriate timing in the assessment of growth hormone secretion and highlight the limitations of relying solely on a single hormone measurement for diagnostic purposes. Notably, in this study all affected participants were male. This observation is consistent with epidemiological data indicating a higher prevalence of GH deficiency in men [46]. Thus, it may suggest that the sex distribution of study populations may influence the reported prevalence of deficiencies of individual pituitary hormones. As noted above, most studies conducted to date on PTHP have been characterized by a predominance of male participants, among whom growth hormone deficiency is epidemiologically more common. Consequently, this may have affected the final study outcomes, which in the great majority pointed to GH deficiency as the most common abnormality.

The study also provides an interesting observation regarding prolactin. The majority of previously published studies have reported hyperprolactinemia as a consequence of TBI [47,48], in line with the hypothesis of dopaminergic pathway injury and subsequent impairment of dopamine-mediated regulatory mechanisms, as well as anatomical damage to the pituitary [48]. In this study, however, contrary to expectations, no significant prolactin elevation was observed. Despite this, after 6 months, a strong, positive, and statistically significant correlation was found between the number of reported symptoms and prolactin levels. It appears that in this case as well, the predominance of women in the current study may have influenced the result. After 6 months, the most common complaint was difficulty concentrating, followed by mood disturbances and mood fluctuations. The obtained findings may be supported by a study on prolactin levels in major depressive disorder, which demonstrated that prolactin levels were higher in women and in patients with major depressive disorder compared with the control group [49]. It appears, however, that the significance of prolactin after TBI has so far been underestimated and represents an area that clearly requires further research. In our study, during the long-term period following injury, higher prolactin levels were correlated with a greater number of adverse symptoms. In contrast, its role in the acute post-brain injury period seems to be beneficial. Studies conducted in patients after non-traumatic brain injury (NTBI) have demonstrated the anti-inflammatory effects of prolactin through glial cell activation, as well as its ability to reduce calcium overload and exert protective effects against excitotoxicity [48]. It would therefore be worthwhile to consider expanding such studies to include patients with traumatic brain injury.

## 5. Limitations

The main limitation of the present study is the small sample size, especially in terms of completing questionnaires, which renders the obtained results indicative of observed trends rather than definitive conclusions and suggests that the study should be considered observational/pilot in nature. Since the study took place in an ED, optimal timing of blood collection for hormonal assays could not always be ensured. Moreover, it was not possible to apply the dynamic tests recommended for the assessment of the somatotropic and corticotropic axes, which limits the strength of the evidence compared with studies employing dynamic tests that are considered the gold standard.

Additionally, concomitant medications taken by the patients, which could potentially have influenced thyroid hormone levels, were not accounted for in the final analysis.

To the best of our knowledge, there is currently no available questionnaire in the literature specifically designed to assess symptoms of hypopituitarism following traumatic brain injury. Existing instruments primarily focus on the quality of life of patients with hypopituitarism. Importantly, the questionnaire developed for the purpose of the present study has not been formally validated. Therefore, the observed symptom–hormone associations should be interpreted with caution and considered hypothesis-generating.

## 6. Conclusions

This pilot study provides an interesting perspective on patient-reported symptoms following mTBI, conceptualizing them as a multifactorial and often difficult-to-classify problem. The findings suggest that post-mTBI symptoms do not necessarily have to be associated with major abnormalities resulting from pituitary damage but may instead result from an overlap between post-concussion symptoms and subtle fluctuations in prolactin and thyroid hormone levels. Importantly, the present study included exclusively patients with mTBI, with women constituting a substantially larger proportion of the study population. In contrast, the majority of previous studies focusing on PTHP, which indicated GH deficiency as the most common hormonal abnormality, predominantly included male patients and individuals with moderate to severe traumatic brain injury. This may suggest the need for future PTHP studies with a more balanced sex distribution among participants to verify whether differences exist between women and men in post-traumatic hormonal disorders, which could in turn imply differences in post-traumatic care.

## Figures and Tables

**Table 1:** Study protocol: schedule of visits and procedures performed at each visit.

Visit No.	Timepoint	Procedures
V_0_	Post-injury	Obtaining consent to participate in the study, inclusion in the study Collecting blood samples for analysis: TSH, fT3, fT4, GH, IGF-1, PRL, ACTH
V_1_	At 3 months	Collecting blood samples for analysis: TSH, fT3, fT4, GH, IGF-1, PRL, ACTH
V_2_	At 6 months	Collecting blood samples for analysis: TSH, fT3, fT4, GH, IGF-1, PRL, ACTH Collecting clinical data and Questionnaire for the Assessment of Symptoms of Anterior Pituitary Insufficiency in Patients After Mild Traumatic Brain Injury (mTBI) Hospitalized in the Emergency Department
V_3_	At 12 months	Collecting blood samples for analysis: TSH, fT3, fT4, GH, IGF-1, PRL, ACTH Collecting clinical data and Questionnaire for the Assessment of Symptoms of Anterior Pituitary Insufficiency in Patients After Mild Traumatic Brain Injury (mTBI) Hospitalized in the Emergency Department

**Table 2:** Patient characteristics.

Clinical Data	Value
Total number of patients (*N*)	56
Female sex, *n* (%)	35 (62.5)
Male sex, *n* (%)	21 (37.5)
Age, median (Q1–Q3), years	45.0 (30.0–61.5)
Positive endocrinological history, *n* (%)	15 (26.8)

**Table 3:** Frequency of symptoms at 6 and 12 months in the completed questionnaires.

Symptom	Frequency at 6 Months (%)	Frequency at 12 Months (%)
Increased fatigue	25	52.6
Pallor	0	5.3
Decreased appetite and weight loss	0	10.5
Cold intolerance	18.8	26.3
Tendency toward constipation	0	21.1
Hoarseness	12.5	10.5
Increased hair loss and dry skin	6.3	26.3
Impaired concentration	62.5	42.1
Decreased libido	6.3	5.3
Mood fluctuations and/or depressive symptoms	37.5	21.1
Loss of hair in the genital area	0	0
Men: loss of facial hair and/or chest hair	0	0
Premenopausal women: menstrual irregularities or hypomenorrhea	0	5.3
Premenopausal women: difficulty conceiving	0	0
Body weight fluctuations	0	15.8
Memory impairment	31.3	36.8
Reduced muscle strength	0	21.1
Abdominal (central) obesity	0	26.3

## Data Availability

All data generated or analyzed during this study are included in this article. Further enquiries can be directed to the corresponding author.

## Supplementary Materials

The following supporting information can be downloaded at https://www.mdpi.com/article/10.3390/diagnostics16060955/s1, File S1: Questionnaire for the Assessment of Symptoms of Anterior Pituitary Insufficiency in Patients After Mild Traumatic Brain Injury (mTBI) Hospitalized in the Emergency Department.

## Abbreviations

ACTH	Adrenocorticotropic hormone
CLIA	Chemiluminescent immunoassay
CMIA	Heterogenous immunochemiluminescence
ESS	Euthyroid sick syndrome
fT3	Free triiodothyronine
GH	Growth hormone
IGF-1	Insulin-like growth factor 1
mTBI	Mild traumatic brain injury
NTIS	Non-thyroidal illness syndrome
PRL	Prolactin
PTHP	Post-traumatic hypopituitarism
T3	Triiodothyronine
TSH	Thyroid-stimulating hormone
TH	Thyroid hormone
