# Supplementary material for: Endocrine Sequelae of Mild Traumatic Brain Injury in Patients Admitted to the Emergency Department: A 12-Month Study
Source: Diagnostics (Basel). 2026 Mar 23;16(6):955. doi: 10.3390/diagnostics16060955 (PMC13026063; doi:10.3390/diagnostics16060955)
Supplement: Supplementary file 1 [file diagnostics-16-00955-s001.zip › diagnostics-4175805-supplementary.pdf]

Questionnaire for the Assessment of Symptoms of Anterior Pituitary Insufficiency in Patients After Mild Traumatic Brain Injury (mTBI) Hospitalized in the Emergency Department

Please indicate whether you have experienced **any of the symptoms below as a result of head injury**.

1. Increased fatigue.
2. Pallor.
3. Decreased appetite and weight loss.
4. Cold intolerance.
5. Tendency toward constipation.
6. Hoarseness.
7. Increased hair loss and dry skin.
8. Impaired concentration.
9. Decreased libido.
10. Mood fluctuations and/or depressive symptoms.
11. Loss of hair in the genital area.
12. **Men:** loss of facial hair and/or chest hair.
13. **Premenopausal women:** menstrual irregularities or hypomenorrhea.
14. **Premenopausal women:** difficulty conceiving.
15. Body weight fluctuations.
16. Memory impairment.
17. Reduced muscle strength.
18. Abdominal (central) obesity.

Source:
The questionnaire was developed based on the following publication:

Mele C, Pingue V, Caputo M, Zavattaro M, Pagano L, Prodam F, Nardone A, Aimaretti G, Marzullo P.
Neuroinflammation and hypothalamo–pituitary dysfunction: Focus on traumatic brain injury.
International Journal of Molecular Sciences. 2021;22:2686. doi:10.3390/ijms22052686
